# Transmission-Type 2-Bit Programmable Metasurface for Single-Sensor and Single-Frequency Microwave Imaging

**DOI:** 10.1038/srep23731

**Published:** 2016-03-30

**Authors:** Yun Bo Li, Lian Lin Li, Bai Bing Xu, Wei Wu, Rui Yuan Wu, Xiang Wan, Qiang Cheng, Tie Jun Cui

**Affiliations:** 1 State Key Laboratory of Millimeter Waves, Southeast University, Nanjing 210096, China; 2 Department of Electronics Engineering and Computer Science, Peking University, Beijing, 100871, China

## Abstract

The programmable and digital metamaterials or metasurfaces presented recently have huge potentials in designing real-time-controlled electromagnetic devices. Here, we propose the first transmission-type 2-bit programmable coding metasurface for single-sensor and single- frequency imaging in the microwave frequency. Compared with the existing single-sensor imagers composed of active spatial modulators with their units controlled independently, we introduce randomly programmable metasurface to transform the masks of modulators, in which their rows and columns are controlled simultaneously so that the complexity and cost of the imaging system can be reduced drastically. Different from the single-sensor approach using the frequency agility, the proposed imaging system makes use of variable modulators under single frequency, which can avoid the object dispersion. In order to realize the transmission-type 2-bit programmable metasurface, we propose a two-layer binary coding unit, which is convenient for changing the voltages in rows and columns to switch the diodes in the top and bottom layers, respectively. In our imaging measurements, we generate the random codes by computer to achieve different transmission patterns, which can support enough multiple modes to solve the inverse-scattering problem in the single-sensor imaging. Simple experimental results are presented in the microwave frequency, validating our new single-sensor and single-frequency imaging system.

The theory and technology of the electromagnetic imaging have been well developed in recent years and many new cutting-edge imaging methods have been proposed, one of which is the well-known single-sensor (or single-radar) imaging^1^. This imaging method is associated with the wavefront reconstruction or inverse scattering problem in the imaging area. The single-sensor system has only one detecting source instead of a source array. To change the modes of the single source, different radiation or receiving patterns are required to reconstruct the objects by solving matrix equations. Thus, the kernel issue in developing the single-sensor imaging system is to generate randomly multiple-mode modulators or masks^2^,^3^,^4^. The masks can also be considered as spatial filters, in which the unit cell’s transmission performance should be varied under the frequency or electronic controls. Recently, an excellent contribution on the single-sensor imaging by using dispersive metamaterials^5^,^6^,^7^,^8^ has been reported by Hunt *et al.*^9^. Then reconfigurable spatial light modulators (SLMs) based on metamaterial absorbers^10^,^11^ have been applied to realize reflection-type single-pixel imaging in the terahertz regime^12^. Sequentially, two-dimensional (2D) holographic metasurfaces have been presented to reconstruct the planar objects^13^,^14^. Combining with the metasurfaces which can flexibly manipulate electromagnetic characteristics of modulators, the single-sensor imaging system will have tremendous potentials and opportunities to promote the imaging performance.

Metasurfaces^15^,^16^,^17^,^18^,^19^,^20^,^21^,^22^,^23^,^24^,^25^ have many advantages over bulk metamaterials, such as the low loss, low cost, and low profile, and hence have overwhelmingly attracted the scientists and engineers in recent years. A number of outstanding results have been presented, including the generalized Snell’s law^18^, Huygens metasurface^21^ and cascaded transmit-array^24^. The above three new concepts or technologies can extremely increase the ability in designing the spatial modulators^26^,^27^, which are the important components for the imaging system and spatial signal processing. The design of spatial modulators using metasurfaces requires that the unit cells with changing texture sizes can overlap 360-degree phase variance under the high transmission rate. For the generalized Snell’s law which introduces the concept of phase discontinuities, the transmission rate is limited to 25% under the cross polarization^27^. For the Huygens metasurfaces, the unit cells are hard to design to satisfy the electric and magnetic resonances under the same frequency. Owing to the high transmission rate and easy realization, the cascaded transmit-array metasurfaces are frequently adopted as the spatial modulators, although they suffer from the thick unit cells composed of multiple layers.

In the imaging area, metamaterials with the negative index of refraction were introduced to realize the perfect image under the ideal case^28^, which opens the door for metamaterial imaging. Based on the theory of amplifying evanescent waves^28^, super- and hyper-lenses have been proposed^29^,^30^,^31^,^32^, which can achieve super-resolution imaging in experiments. Distinguished from the super/hyper-lens approaches^28^,^29^,^30^,^31^,^32^, metamaterials or metasurfaces have also contributions in the wave-front reconstruction or calculation imaging. More recently, the holographic imaging has rapidly developed by using metasurfaces^33^, which can generate arbitrary optical images with different recordings of the hologram. Ni *et al.* presented the thinnest hologram to modulate both amplitudes and phases in the visual band, which produces high-resolution and low-noise images with the aids of complementary V-type nano-antennas^34^. Simultaneously, by using the plasmonic metasurfaces, Zhang *et al.* have reconstructed images of complex objects in the optical band with eliminating the undesired effects of multiple diffractions^35^, and also made the meta-hologram reach 80% conversion efficiency between two circular-polarization states^36^.

More recently, coding, digital, and programmable metamaterials have been presented^37^,^38^, providing a new way to describe metamaterial. In the reflection-type 1-bit programmable metamaterial^37^, the unit cells were controlled by the field programmable gate array (FPGA) to reach zero reflection phase (“0” state) or 180-degree reflection phase (“1” state). Then the reflections and scattering of electromagnetic waves can be manipulated by changing the “0” and “1” states of each unit cell in real time by FPGA, realizing instant switch among different functionalities, such as single-beam reflection, multi-beam scattering, and diffusions^37^. However, transmission-type programmable metamaterial has not been reported yet, which has more application potentials.

In this article, we propose a novel transmission-type 2-bit programmable metasurface and then present a single-sensor and single-frequency imaging system in the microwave frequency, which makes use of only one sensor with multi-mode measurements to reconstruct the object. In our design, the coding unit contains two layers, which is convenient for changing the voltages in rows and columns to switch the diodes in the top and bottom layers. We use computer to manipulate FPGA to deliver the random binary codes “0” and “1” for switching the diode states on the unit cells, which results in the change of transmission features of the whole metasurface. Due to the dyadic states in each layer of the unit cell, the whole aperture can be made 2-bit coding programmable. For the transmission performance of the 2-bit units, we need enough phase variety between coding units to keep high transmission amplitudes. Then, excited by a single horn antenna (i.e. the single sensor), the 2-bit programmable metasurface can randomly modulate and generate many different transmission patterns, which can provide adequate modes to solve the inverse scattering problem in the microwave imaging system. Compared with the recently excellent work for terahertz single-pixel imaging based on SLMs^12^, we can control the rows and columns of the metasurface aperture simultaneously using the 2-bit programmable coding metasurface, instead of tuning all unit cells independently^12^. For instance, for an *N* × *N*-element metasurface, we need only 2*N* random controls to the row and column, instead of *N*^2^ independent controls to all elements^12^, which will reduce the costs and complexities of the imaging system significantly. Compared with the pioneer work on single-sensor imaging methods to transform the masks controlled by frequency using dispersive and resonance metasurfaces^9^,^13^,^14^, the proposed programmable imaging system with variable modulators under single frequency can avoid the object dispersion.

## Results and Discussions

**Figure 1:**
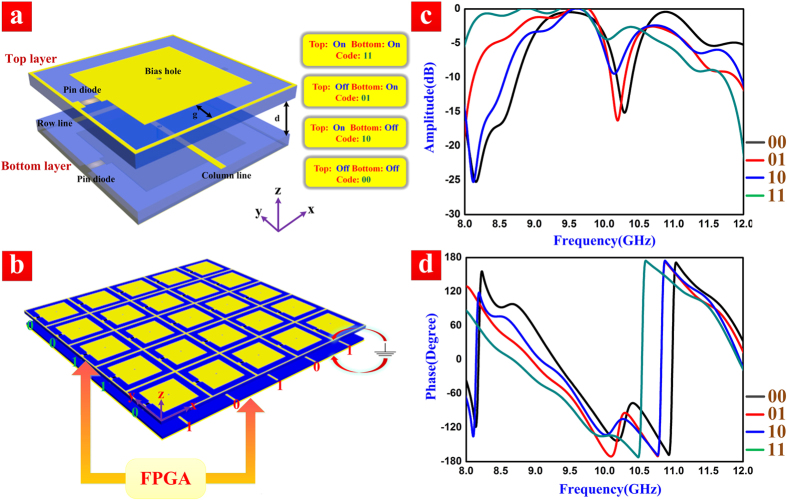
**(a)**The diagram of the 2-bit coding unit cell. The period of the unit is 14 mm, and a commercial printed circuit board F4B is chosen as the dielectric substrate (blue areas) with the relative permittivity of 2.2 and the thickness of 1 mm. The texture has two layers: the top layer and the bottom layer, which have mirror-symmetrical geometries except of the feeding lines. The two layers are separated by air with the gap of d = 3 mm. The pin diodes are connected between the fishnets in the outer rings and the middle metallic patches, which are insulated by the size of g = 2 mm. **(b)** The 2-bit programmable coding metasurface for our single-sensor and single-frequency imaging, in which FPGA can randomly deliver the binary codes (0 or 3.3 V voltage) on rows (green) and columns (red), simultaneously. **(c,d)** The simulated amplitudes **(c)** and phases **(d)** of transmission characteristics of the 2-bit coding unit cell with different states.

To realize the single-sensor and single-frequency imaging system, the core task is to propose the programmable spatial modulators controlled by digital codes. We choose here the transmit-array metasurface with high transmission amplitude and large phase variety as our spatial modulators. The unit cell of the programmable metasurface has two layers, as shown in Fig. 1(a), in which each layer contains a switchable diode. For conveniently grounding, we use a metallic fishnet texture^39^ in the edges of the unit cell. The positive voltages are loaded through via holes. On the top layer of the unit cell, the back feeding line is horizontal; while on the bottom layer, the back feeding line is vertical, which will perform the controls of rows and columns, respectively. We choose a commercially available “SMP 1320-079LF” as the switchable diode, whose parameters are introduced in the legend of Fig. 1. When positive voltage is loaded though via hole, we define the state of the diode as “On”; when no voltage is loaded, the state is defined as “Off”. Thus, according to the structure of the two-layer unit cell, there are four states in the whole texture due to the dual responses in each layer. We indicate the four states as 2-bit binary codes: “11”, “01”, “10”, and “00”, respectively.

Here, we want to reconstruct the objects under a single frequency between 9 to 10 GHz. For this purpose, we first extract the parameters of the pin diode in this frequency band. An “R-L-C” series model is assumed and the extracted results are R = 0.06 Ω, L = 0.45 nH, and C = 0.25 pF when the loaded voltage is 0 V; and R = 0.17 Ω, L = 0.89 nH, and C = 2.75 pF when the loaded voltage is 3.3 V. By importing the “R-L-C” parameters into the commercial software, CST Microwave Studio, we can simulate the transmission performance of the unit cell (S21), as shown in Fig. 1(c,d). Here, different codes mean different states of the Pin diodes in the upper and lower layers of the unit cell. Hence the current distributions of the unit cell under different codes are varied, which lead to affect the transmission coefficients. The parameter sweeping method is used to design the 2-bit unit cells based on the full-wave simulations by loading the lumped circuit. The simulation results indicate that the 2-bit coding unit has good features of high-amplitude transmissions and enough phase shifts in the considered frequency band, which satisfy the requirements to our single-sensor imaging. Based on the unit-cell design, we create a 5 × 5 transmission-type programmable metasurface, as illustrated in Fig. 1(b). The thickness of the metasurface is 5 mm, which is close to *λ*/7 (λ is the wavelength at 9 GHz). We use FPGA to deliver the randomly binary codes, which control the high and zero voltages on the row and column lines to generate the 2-bit coding. Though each metasurface unit cell is not controlled individually, the row and column of the metasurface are manipulated simultaneously so that all unit cells possessing on their own spatial positions have independent 2-bit random codes.

**Figure 2:**
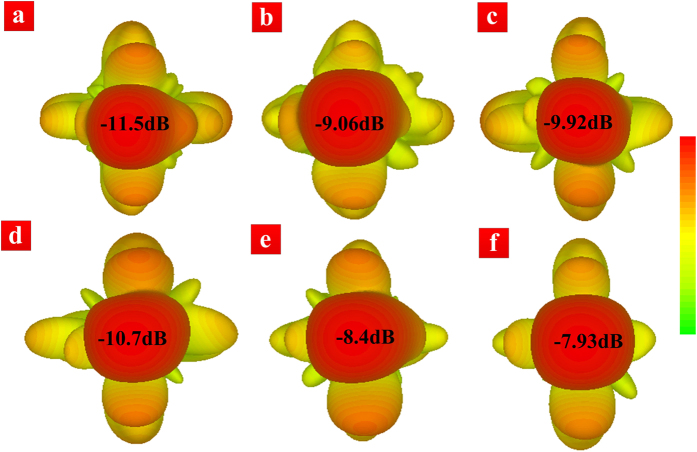
The transmission patterns (i. e., forward radar cross sections) in the far-field region with different programmable scenarios at 9.2 GHz, in which the values of relative transmission gains are given. (**a**) Row codes: 00000; Column codes: 10000. (**b**) Row codes: 01100; Column codes: 10110. (**c**) Row codes: 00001; Column codes: 11001. (**d**) Row codes: 10000; Column codes: 10001. (**e**) Row codes: 11001; Column codes: 11100. (**f**) Row codes: 11101; Column codes: 10111.

In fact, we can generate many groups of “row and column” binary codes to randomly modulate the masks of the transmission-type programmable metasurface. Figure 2(a–f) illustrate some selected transmission patterns through the metasurface obtained by full-wave numerical simulations. From Fig. 2(a–f), we find that the appearances and gains of the transmission patterns are both discriminative under different programmable modulations. The results clearly demonstrate that, by using the proposed method (controlling “row and column” simultaneously) to manipulate the metasurface masks, we can obtain many different transmitting and receiving patterns, which are the essential for the single-sensor image reconstruction.

For the single-sensor and single-frequency imaging system, we need many different measurement modes, and thus many different metasurface patterns, to constitute a generalized system response matrix, which characterizes the signal sensed by the horn antenna for a given configuration of metasurface illuminated by the same horn antenna. The actual measurement signals under a given metasurface mode is related to the original object-area vector *σ* = (*σ*_*i*_), *i* = 1, 2, …, *N*, through the generalized system response matrix ***G*** = (*G*_*pj*_), *p = 1, 2*, …*, P, j* = 1, 2, …, *N*. The relationship amongs *σ*, *G* and the measurement data ***V*** = (*V*^(*p*)^_*i*_), *p* = 1, 2, …, *P*, is expressed as


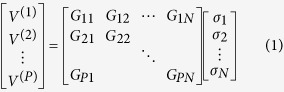


**Figure 3:**
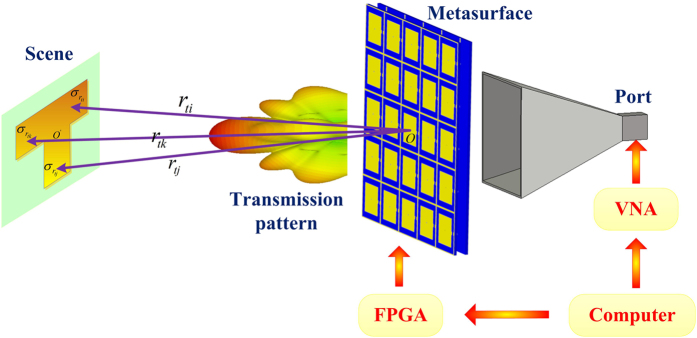
The schematic of the proposed single-sensor and single-frequency microwave imaging system, in which the horn antenna is considered as the single sensor, while the vector network analyzer (VNA) can be treated as the transmitter and receiver.

To interpret the establishment of Eq. (1), as shown in Fig. 3, *O* is the original point of global coordinate system, which is the central point of the programmable metasurface aperture as well, and *O*′ is the central point of the image scene. For the metasurface with the *p*th pattern (*p* = 1, 2, … *P*, *P* indicates the total number of metasurface patterns for the whole imaging) configured by FPGA, the electrical field radiated from the horn antenna after experiencing through the metasurface reads^37^:


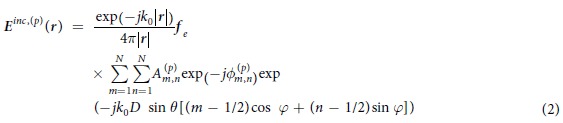


The quantities 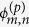 and 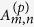 correspond to the phase and amplitude distributions, respectively, which are caused by the illumination of horn antenna, over the (*m,n*)th metasurface unit with the *p*th metasurface configuration. It remains an open and challenging problem to determine 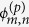 and 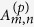 with the acceptable accuracy for the imaging purpose because of the really complicated interactions among the metasurface elements and the microwave signal diffracted from the horn antenna. The summation involved in Eq. (2) is performed across the whole radiation elements of the metasurface. Other parameters introduced in Eq. (2) are explained as follows, i.e., ***f***_*e*_ denotes the radiation pattern of unit, *k*_0_ is the operational wavenumber in free space, *D* is the size of the element and *θ* and *φ* are associated with polar and azimuth angles of ***r***, respectively. For notable convenience, Eq. (2) is formally rewritten as


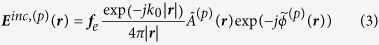


With such illumination as descripted by Eq. (2) or Eq. (3), the current ***J***_*s*_(**r**′) induced on the surface of the conducting object is


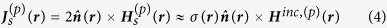


Herein, 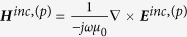, 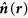 is the normal direction on the conducting object surface, and σ(**r**′) = 2 is associated with the conducting pixel, and being zero otherwise. It is noted that the geometrical optical approximation has been explicitly made for the smooth object in the second equality of Eq. (4). Now, following the standard Huygens’s principle, we can immediately arrive at the scattered electrical field emerged from the probed object illuminated by Eq. (2), namely,


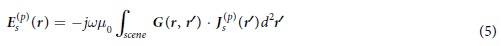


Herein, the dyadic Green’s function reads


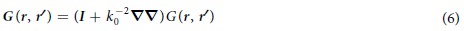


where 
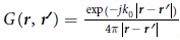
 is the scalar Green’s function in free space.

With the use of the reciprocal property, the signal sensed by the horn antenna is:


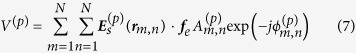


Combing Eqs (2, 3, 4, 5) with Eq. (7) leads to following linear expression relating the observation *V*^(*p*)^ and the reflectivity of probed object σ(**r**′), namely,


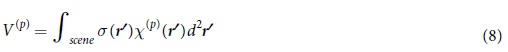


where


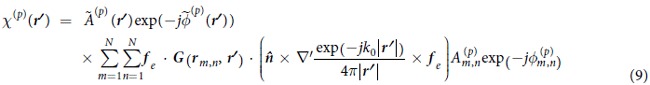


For the purpose of numerical operation, the image scene is evenly divided into *N* sub-areas, in each the sub-RCS is defined as σ_*j*_(*j* = 0, 1…*N*). In order to avoid introducing too many notations, *G*_*pj*_(*p* = 0,1…*P*; *j* = 0, 1…*N*), calculated from Eq. (9), is used to represent the system response relating the *p*th observation and the *j*th pixel. Then, Eq. (8) can be cased into the compact form (Eq. (1)) after stacking all *P* measurements corresponding to *P* metasurface patterns.

In our design, both P and *N* are equal to 5 × 5 = 25 and the matrix ***G*** is composed of 25 × 25 = 625 units. Eq. (1) can be rewritten as ***V*** = ***Gσ*** for short. Hence it is obvious that ***σ*** = ***G***^−1^***V*** when the matrix ***G*** is a square matrix. That is to say, if the object area to be reconstructed has 25 sub-object areas, the times of measurements must be 25 for solving the unknown sub-objects by using the directly inverse operation. Thus, the main purpose of the multi-times measurements under different programmable transmission modes is to build up the matrix equation.

According to the theory of single-sensor imaging, we present the flow chart of the whole imaging system, as shown in Fig. 3. Here, the computer is the brain of the imaging system, which can control the vector network analyzer (VNA) and FPGA to further control the single source (i.e., the horn antenna) and the transmission-type programmable metasurface, respectively. We set the object to be reconstructed in the far-field region. First, the computer emits the random “row and column” binary codes stored in FPGA to complete the mask modulations. Then, the computer sends the orders of transmission and receiving to VNA. This situation can be considered as the single-times measurement in the imaging system. To build up the matrix equation, we should make 25 times (in this design) measurements under different programmable coding patterns at the same frequency. However, the unknown generalized system response matrix should be calibrated before the imaging experiments. To distinguish with the former metamaterial-based single-sensor imaging system, we apply the pin diodes controlled by voltages to change the transmission performance of the metasurface at single frequency, instead of using the dispersive characteristics of metasurfaces to avoid the object dispersion.

To further validate the new method of the single-sensor and single-frequency imaging system, we fabricate a sample of the transmission-type programmable metasurface and complete the imaging experiments. The sample is composed of 10 × 10 = 100 elements with the size of 140 × 140 mm^2^. However, in our experiment we can only use 5 × 5 = 25 elements in the center of the metasurface, which are efficiently illuminated by the horn antenna. Sufficiently different (or random) transmission patterns for the imaging system are generated by using the programmable coding elements. Considering to the exclusive calibration time of the generalized system response matrix, we only divide the object area to be solved into 25 sub-areas, and the size of each sub-area is 20 × 20 mm^2^ (the resolution is about 2*λ*/3 at 9 GHz). More elements of the sample should be used if we want to reconstruct a bigger field of view (FOV) with high efficiency.

**Figure 4:**
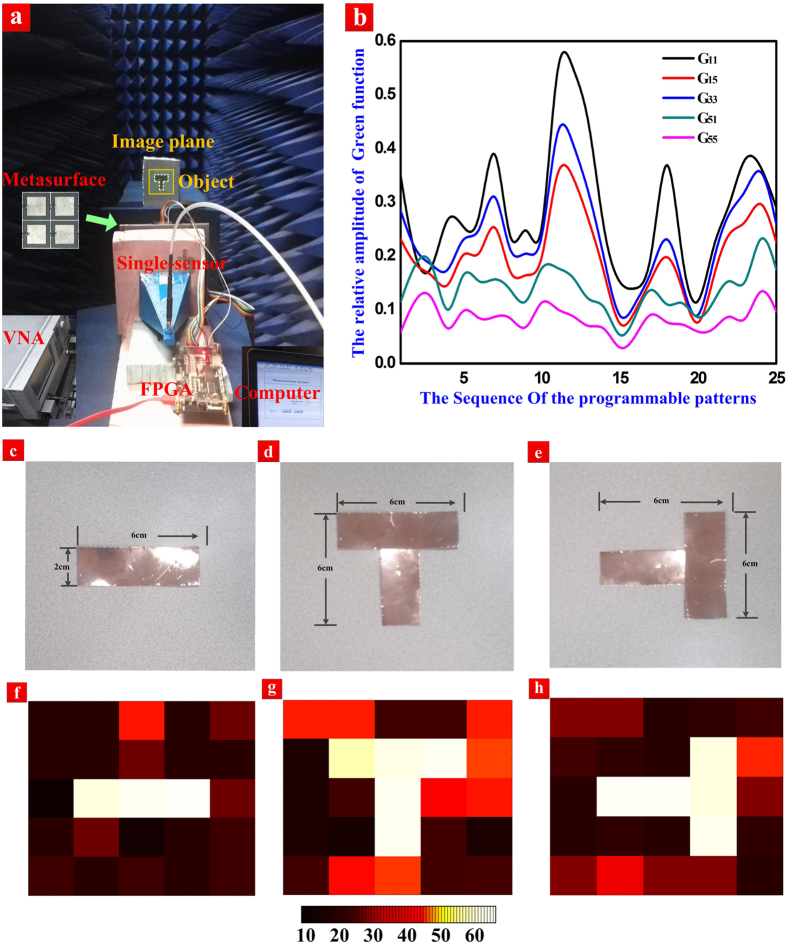
**(a)** The single-sensor and single-frequency imaging system based on the 2-bit programmable coding metasurface. **(b)** The sampled information of the generalized system response matrix (G11, G15, G33, G51, and G55) calibrated under the 25 different programmable patterns in our measurements. For each randomly varied curve, it can prove that the diodes are working in the experiments. **(c–e)** The photographs of three metallic objects: the horizontal bar **(c)**, the T-type object **(d),** and the T-type object rotated by 90 degrees **(e)**. **(f–h)** The imaging results of the three metallic objects at 9.2 GHz: the horizontal bar **(f)**, the T-type object **(g)**, and the T-type object rotated by 90 degrees **(h)**.

It is very important to calibrate the generalized system response matrix with regard to the imaging system by changing the transmission patterns. There exist two methods to get such matrix: 1) Acquire the near-filed currents on the imaging aperture, and then the far-filed transmission and receiving patterns will be obtained by applying the spatial Fourier transform; 2) Move a regular small metallic object for calibration in all sub-areas on the imaging plane to obtain the transmission and receiving performance in the manner of point-to-point scanning, which are the information of the system response function. According to the practicability, we choose the second method to make calibration, but the calibration process is complicated and time-consuming. The Labview software is applied to connect VNA (Agilent N5230C) and FPGA to the computer, which is also used to control the transmission and sampling of signals. We adopt the technology of background cancellation and loading the hardware time-domain gate to eliminate the effect of background noise. The intermediate frequency bandwidth is reduced for filtering the random noise in experiments. Figure 4(b) shows some calibration results of the generalized system response matrix under the change of coding patterns.

From Fig. 4(b), we observe that the generalized system response matrix in each sub-area has irregular (or random) variety under different programmable coding patterns, and thus it is appropriate for reconstructing the object based on our imaging system. In experiments shown in Fig. 4(a), we have reconstructed three metallic objects: a horizontal bar, a T-type object, and a T-type object rotated by 90 degrees, as displayed in Fig. 4(c–e). The experimental imaging results at 9.2 GHz are presented in Fig. 4(f–h), which clearly validates the efficiency of the single-sensor and single-frequency imaging system.

## Conclusions

We have proposed a new transmission-type 2-bit programmable coding metasurface and further built up a single-sensor and single-frequency microwave imaging system. In design of the imaging system, we have realized to change the masks randomly using the programmable metasurface, in which the rows and columns can be controlled simultaneously by the binary codes. Compared to the conventional methods of single-sensor imaging, in which all unit cells are manipulated independently, the proposed imaging system can greatly reduced the cost and complexity of the feeding circuits. Because it is based on the single-frequency reconstruction without the frequency agility, this imaging system also avoids the object dispersion. The 2-bit programmable coding metasurface is composed of transmission-type elements to realize many different transmission patterns, which are the essential parts for solving the inverse problem in the single-sensor and single-frequency imaging. The proposed method is not only applicable for the far-filed imaging, but also for the near-field imaging, which will be studied in our future work. The new imaging system may also be extended to the terahertz frequencies.

## Additional Information

**How to cite this article**: Li, Y. B. *et al.* Transmission-Type 2-Bit Programmable Metasurface for Single-Sensor and Single-Frequency Microwave Imaging. *Sci. Rep.* **6**, 23731; doi: 10.1038/srep23731 (2016).

## References

[b1] Duarte M. F. *et al.* Single-pixel imaging via compressive sampling. IEEE Signal Process. Mag 25, 83 (2008).

[b2] Chan W. L. *et al.* a spatial light modulator for terahertz beams. Appl. Phys. Lett. 94, 213511 (2009).

[b3] Watts C. M., Liu X. & Padilla W. J. Metamaterial electromagnetic wave absorbers. Adv. Opt. Mater. 24, 98 (2012). 10.1002/adma.201200674 22627995

[b4] Sensale-Rodriguez B. *et al.* Terahertz imaging employing graphene modulator arrays. Opt. Express. 21, 2324 (2013). 23389211 10.1364/OE.21.002324

[b5] Smith D. R., Pendry J. B. & Wiltshire M. C. K. Metamaterials and Negative Refractive Index. Science 305, 788 (2004). 15297655 10.1126/science.1096796

[b6] Schurig D. *et al.* Metamaterial Electromagnetic Cloak at Microwave Frequencies. Science 314, 977 (2006). 17053110 10.1126/science.1133628

[b7] Lai Y., Chen H. Y., Zhang Z. Q. & Chan C. T. Complementary Media Invisibility Cloak that Cloaks Objects at a Distance Outside the Cloaking Shell. Phys. Rev. Lett. 102, 093901 (2009). 19392518 10.1103/PhysRevLett.102.093901

[b8] Luo Y., Zhang J. J. B., Wu I. & Chen H. S. Interaction of an electromagnetic wave with a cone-shaped invisibility cloak and polarization rotator. Phys. Rev. B 78, 125108 (2008).

[b9] Hunt J. *et al.*, Metamaterial apertures for computational imaging. Science 339, 310 (2013). 23329043 10.1126/science.1230054

[b10] Landy N. I., Sajuyigbe S., Mock J. J., Smith D. R. & Padilla W. J. Perfect metamaterial absorber. Phys. Rev. Lett. 100, 207402 (2008). 18518577 10.1103/PhysRevLett.100.207402

[b11] Chen H. T. *et al.* Antireflection coating using metamaterials and identification of its mechanism. Phys. Rev. Lett. 105, 073901 (2010). 20868044 10.1103/PhysRevLett.105.073901

[b12] Watts C. M. *et al.* Terahertz compressive imaging with metamaterial spatial light modulators. Nat. Photonics. 8, 605 (2014).

[b13] Lipworth G. *et al.* Metamaterial apertures for coherent computational imaging on the physical layer. J. Opt. Soc. Am. A 30, 1603 (2013). 10.1364/JOSAA.30.001603 24323219

[b14] Hunt J. *et al.* Metamaterial microwave holographic imaging system. J. Opt. Soc. Am. A 31, 2109 (2014). 10.1364/JOSAA.31.002109 25401233

[b15] Holloway C. L. M., Mohamed A., Kuester E. F. & Dienstfrey A. Reflection and transmission properties of a metafilm: With an application to a controllable surface composed of resonant particles. IEEE Trans. Antennas Propag. 47, 853 (2005).

[b16] Holloway C. L. *et al.* An Overview of the Theory and Applications of Metasurfaces: The Two-Dimensional Equivalents of Metamaterials. IEEE Trans. Antennas Propag. 54, 10 (2012).

[b17] Sun S. *et al.* Gradient-index meta-surfaces as a bridge linking propagating waves and surface waves. Nat. Mater. 11, 426 (2012). 22466746 10.1038/nmat3292

[b18] Yu N. *et al.* Light Propagation with Phase Discontinuities: Generalized Laws of Reflection and Refraction. Science 334, 333 (2011). 21885733 10.1126/science.1210713

[b19] Yu N. & Capasso F. Flat optics with designer metasurfaces. Nat. Mater. 13, 139 (2014). 24452357 10.1038/nmat3839

[b20] Liu L. X. *et al.* Broadband Metasurfaces with Simultaneous Control of Phase and Amplitude. Adv. Mater. 26, 5031 (2014). 24863731 10.1002/adma.201401484

[b21] Pfeiffer C. & Grbic A. Metamaterial Huygens’ Surfaces: Tailoring Wave Fronts with Reflectionless Sheets. Phys. Rev. Lett. 110, 197401 (2013). 23705738 10.1103/PhysRevLett.110.197401

[b22] Germain D., Seetharamdoo D., Burokur S. N. & De Lustrac A. Phase-compensated metasurface for a conformal microwave antenna. Appl. Phys. Lett. 103, 124102 (2013).

[b23] Li Y. B., Wan X., Cai B. G., Cheng Q. & Cui T. J. Frequency-controls of electromagnetic multi-beam radiations and beam scanning by metasurfaces. Sci. Rep. 4, 6921 (2014). 25370447 10.1038/srep06921 PMC4220278

[b24] Pfeiffer C. & Grbic A. Cascaded metasurfaces for complete phase and polarization control. Appl. Phys. Lett. 102, 231116 (2013).

[b25] Li Y. B., Cai B. G., Wan X. & Cui T. J. Diffraction-Free Surface Waves by Metasurfaces, Opt. Lett. 39, 5888 (2014). 25361111 10.1364/OL.39.005888

[b26] Jiang S. *et al.* Controlling the Polarization State of Light with a Dispersion-Free Metastructure. Phys. Rev. X. 4, 041042 (2014).

[b27] Monticone F., Estakhri N. M. & Alu A. Full Control of Nanoscale Optical Transmission with a Composite Metascreen. Phys. Rev. Lett. 110, 203903 (2013). 25167411 10.1103/PhysRevLett.110.203903

[b28] Pendry J. B. Negative Refraction Makes a Perfect Lens, Phys. Rev. Lett. 85, 3966 (2000). 11041972 10.1103/PhysRevLett.85.3966

[b29] Fang N., Lee H., Sun C. & Zhang X. Sub–Diffraction-Limited Optical Imaging with a Silver Superlens. Science 308, 534 (2005). 15845849 10.1126/science.1108759

[b30] Zhang X. & Liu Z. Superlenses to overcome the diffraction limit. Nat. Mater. 7, 435 (2008). 18497850 10.1038/nmat2141

[b31] Jiang W. *et al.* Broadband All-Dielectric Magnifying Lens for Far-Field High-Resolution Imaging. Adv. Mater. 25, 6963 (2013). 24352983 10.1002/adma.201303657

[b32] Ma C. & Liu Z. A super resolution metalens with phase compensation mechanism. Appl. Phys. Lett. 96, 183103 (2010).

[b33] Lee W. H. Sampled Fourier Transform Hologram Generated by Computer. Appl. Opt. 9, 639 (1970). 20076253 10.1364/AO.9.000639

[b34] Ni X., Kildishev A. V. & Shalaev V. M. Metasurface holograms for visible light. Nat. Commun. 4, 2807 (2013).

[b35] Huang L. *et al.* Three-dimensional optical holography using a plasmonic metasurface, Nat. Commun. 4, 2808 (2013).

[b36] Zheng G. *et al.* Metasurface holograms reaching 80% efficiency. Nat. Nanotech. 10, 308 (2015). 10.1038/nnano.2015.2 25705870

[b37] Cui T. J., Qi M. Q., Wan X., Zhao J. & Cheng Q. Coding metamaterials, digital metamaterials and programmable metamaterials. Light: Sci. & Appl. 3, e218 (2014).

[b38] Giovampaola C. D. & Engheta N. Digital metamaterials. Nat. Mater. 13, 1115 (2014). 25218061 10.1038/nmat4082

[b39] Kafesaki M. *et al.* Left-handed metamaterials: The fishnet structure and its variations. Phys. Rev. B 75, 235114 (2007).

